# Comparative Transcriptome Profiling Provides Insights into Plant Salt Tolerance in Watermelon (*Citrullus lanatus*)

**DOI:** 10.3390/life12071033

**Published:** 2022-07-12

**Authors:** Yingchun Zhu, Gaopeng Yuan, Bowen Gao, Guolin An, Weihua Li, Wenjing Si, Dexi Sun, Junpu Liu

**Affiliations:** Zhengzhou Fruit Research Institute, The Chinese Academy of Agricultural Sciences, Zhengzhou 450009, China; zhuyingchun@caas.cn (Y.Z.); yuangaopeng@caas.cn (G.Y.); gaobowen0514@163.com (B.G.); anguolin@caas.cn (G.A.); liweihua@caas.cn (W.L.); siwenjingsmile@sina.cn (W.S.)

**Keywords:** comparative transcriptome, salt tolerance, watermelon, molecular mechanism, TCP

## Abstract

Salt stress seriously reduced the yield and quality of watermelon and restricted the sustainable development of the watermelon industry. However, the molecular mechanism of watermelon in response to salt stress is still unclear. In this study, 150 mmol·L^−1^ NaCl was used to deal with the seedlings of salt-tolerant and salt-sensitive watermelon varieties. Physiological characteristics showed that salt stress significantly reduced the biomass of watermelon seedlings and the accumulation of K^+^ in roots and leaves and significantly increased the content of Na^+^, Cl^−^, and malondialdehyde (MDA). Compared with the salt-sensitive variety, the salt-tolerant variety had higher K^+^ accumulation, lower Cl^−^, Cl^−^ accumulation, and MDA content in roots and leaves. Then, RNA-seq was performed on roots and leaves in normal culture and under 150 mmol·L^−1^ NaCl treatment. A total of 21,069 genes were identified by RNA-seq analysis, of which 1412 were genes encoding transcription factors (TFs). In the comparison groups of roots and leaves, 122 and 123 shared differentially expressed genes (DEGs) were obtained, respectively. Gene ontology (GO) annotation and KEGG enrichment results showed that there were many identical GO terms and KEGG pathways in roots and leaves, especially the pathways that related to sugar or energy (ATP or NADP^+^/NADPH). In addition, some DEGs related to salt tolerance were identified, such as plant hormone indole-3-acetic acid (IAA) and gibberellin (GA) signal transduction pathway-related genes, K^+^/Na^+^/Ca^2+^-related genes, lignin biosynthesis-related genes, etc. At the same time, we also identified some TFs related to salt tolerance, such as AP2-EREBP, bZIP, bHLH, MYB, NAC, OFP, TCP, and WRKY and found that these TFs had high correlation coefficients with salt tolerance-related genes, indicating that they might have a potential regulatory relationship. Interestingly, one TCP TF (Cla97C09G174040) co-exists both in roots and leaves, and it is speculated that it may be regulated by *miR319* to improve the salt tolerance of watermelon.

## 1. Introduction

Salt stress is an important factor affecting the sustainable development of global agriculture. Around 1 billion hm^2^ of land worldwide are affected by salt stress, and soil salinization is expected to reach more than 50 percent of global arable land by mid-century [1]. Salt stress can cause primary stresses such as osmotic stress and ion toxicity, and high salt can also cause a series of secondary stresses such as oxidative stress and nutritional stress [2]. The accumulation of multiple stresses will affect cell growth and metabolism, thereby affecting seed germination, seedling growth, and crop yield [3,4]. In order to increase survival opportunities, plants have evolved complex mechanisms to cope with salt stress at the morphological structure, physiological metabolism, and molecular level, including the reduction of leaf number and leaf area, stomatal closure, accumulation of osmotic adjustment substances, efflux and compartmentalization of Na^+^ and Cl^−^, removal of reactive oxygen species (ROS), and changes in stress-responsive gene expression [1].

Ion metabolic balance plays an important role in maintaining cell membrane stability and ensuring plant growth and development [5]. Excessive accumulation of Na^+^ and Cl^−^ is the main cause of salt damage, resulting in a lack of Ca^2+^ and K^+^ and other important ions. The content of Na^+^ in plants increased with the increase of Na^+^ concentration in soil [6]. Under the action of a chemical gradient, the decrease of membrane potential caused by high concentration of Na^+^ can promote the absorption of Cl^−^. Excessive Na^+^ ions can cause metabolic disorders and change the function of some enzymes [7]. High concentration of Na^+^ will also leads to cell osmotic pressure imbalance, loss of membrane function, and increase of ROS, thereby affecting the normal growth of plants [8]. In addition, Cl^−^ can destroy the cell membrane system and organelle structure, resulting in a decrease in chlorophyll content, which is not conducive to plant growth [4]. Except for the toxic effect of Na^+^, its ionic radius and hydration energy are similar to those of K^+^, which shows obvious competitive inhibition on K^+^. Normally, relatively high K^+^/ Na^+^ ratio maintained in the cytoplasm ensures cell physiological activity. However, the influx of a large number of external Na^+^ under salt stress will inhibit the absorption of K^+^, resulting in damage to K^+^ deficiency in plants. Similarity, intracellular Ca^2+^ levels are also reduced by competitive inhibition of Na^+^. For example, the Ca^2+^/Na^+^ ratio of *Schizonepeta tenuifolia* decreased with the increase of NaCl concentration [9]. Furthermore, salt stress usually causes osmotic stress and cell membrane dysfunction, and expansion pressure, osmotic pressure, relative water content (RWC), MDA, and ROS will change with salt stress [10]. The cell membrane plays an important role in material transport, energy transfer, and signal transduction, and its selective permeability enables it to regulate ion homeostasis and ensure plant physiological activities. However, excessive ROS can cause great damage to the membrane under salt stress, mainly reflected in its influence on ion selectivity, flow rate, and transport, and lead to a large number of electrolyte leakages, resulting in osmotic pressure stress, increasing the relative permeability of the membrane and reducing the fluidity of the membrane [5].

In production, salt tolerance of plants is generally improved by applying exogenous chemicals, including substances that can activate plant antioxidant enzyme activities, such as NO, silicon, osmotic regulators such as betaine, sugar, organic acids, and other substances, naphthaleneacetic acid (NAA), jasmonic acid (JA), GA, and other plant growth regulators. In addition, salicylic acid (SA), humic acid, and Ca^2+^ can reduce the permeability of the cell membrane [1]. However, these exogenous substances will not fundamentally solve the problem of salt stress. Introducing salt tolerance-related genes into plants by genetic engineering is the simplest and most efficient way to improve salt tolerance.

The planting area of watermelon (*Citrullus lanatus*) in China is more than 1 million hm^2^, which makes watermelon one of the main melon and fruit crops. Watermelon roots are weak and sensitive to salt stress [11]. Previous study had shown that the growth of seedlings is inhibited when the salt concentration is greater than 75 mmol·L^−1^. The stress degree of watermelon seedlings increased with the increase of salt concentration, and the growth and development of seedlings are strongly inhibited when the salt concentration is greater than 150 mmol·L^−1^, which seriously reduces the yield and quality of watermelon and restricts the sustainable development of the watermelon industry [12]. It has grown to be an urgent problem to alleviate the effect of salt damage on watermelon. Therefore, it is of great significance to explore the salt tolerance mechanism and identify the salt tolerance genes for the breeding of new varieties of salt-tolerant watermelon and the healthy development of watermelon industry. In this study, salt-tolerant and salt-sensitive watermelon varieties were used as experimental materials. Firstly, physiological indexes of watermelon seedlings before and after 150 mmol·L^−1^ NaCl treatment were determined to clarify their tolerance to salt stress. Then, RNA-seq analysis was carried out on the leaves and roots of the two varieties to analyze the transcriptional differences of genes under salt stress, and the candidate genes related to salt tolerance of watermelon were excavated to provide scientific basis for new varieties breeding of salt-tolerant watermelon.

## 2. Materials and Methods

### 2.1. Plant Materials and Treatments

The seeds of the salt-tolerant watermelon variety (‘Zhongshihong’) and the salt-sensitive watermelon variety (‘PI186489’) [13] were sown in clay after germination and then transplanted to Hoagland solution after cotyledons were fully expanded and grown in 16 h of light/8 h of dark. After 30 days of growth, seedlings were cultured in Hoagland solution containing 150 mmol·L^−1^ NaCl in intelligent greenhouses (25 °C, 16 h of light/8 h of dark) (Supplementary Figure S1). Roots of seedlings were sampled after 7 days of treatment. Some of the samples were stored at −20 °C for physiological and biochemical index determination. The other part was equally divided into two parts and the seedlings were frozen in liquid nitrogen immediately for RNA extraction. There were 30 seedlings of each treatment; when sampling, each sample contained 10 seedlings, with 3 biological replicates per treatment.

### 2.2. Measurements of Physiological and Biochemical Index

The biomass was the dry weight of the whole plant. Firstly, the surface of the seedling was dried with filter paper, and the seedling was dried in an oven until constant weight and then weighed dry weight.

The MDA content was determined according to Yuan et al. [14] using a KTB1050, (Abbkine, Beijing, China) kit, and then the absorbance values at 532 nm and 660 nm were determined, respectively. The Na^+^, Cl^−^, and K^+^ content was determined using a sodium assay kit (C002-1-1), chlorine assay kit (C003-2-1), and potassium assay kit (C001-2-1) (Nanjing Jiancheng Bioengineering Institute, China), and the wavelength of Na^+^, Cl^−^, and K^+^ was 620 nm, 480 nm, and 450 nm, respectively. SpectraMax (Molecular Devices, China) was used to measure absorbance.

All data were statistically analyzed using Office Excel 2016 software. SPSS 18.0 software was used to sort out the data for one-way ANOVA statistical analysis, and the significant difference was defined as *p* < 0.05 (*n* = 3).

### 2.3. RNA Extraction, Sequencing and Expression Profiling

Total RNA was isolated from the roots and leaves of 24 samples. Firstly, RNA was extracted using RNeasy Plant Mini Kit (Beijing Tiangen) according to the manufacturer’s instructions. Secondly, total RNA was qualified and quantified using a Nano Drop and Agilent 2100 bioanalyzer (Thermo Fisher Scientific, Waltham, MA, USA). Thirdly, high quality and more than 200 ng RNA were amplified and reversely transcribed to cDNA based or polyA tail. The template was switched to the 5’ end of RNA and full-length cDNA was generated by PCR. The cDNA fragments verified and purified in the previous step were segmented by PCR. The cDNA was quantitatively analyzed by an Agilent 2100 Bioanalyzer. The double-chain PCR product was thermally denatured and cycled by splinting oligonucleotide sequences through quality check steps. At last, single-strand circle DNA (ssCir DNA) was formatted into the final library. The final library was phi29 (Thermo Fisher Scientific, USA), which manufactured DNA nanospheres (DNB) by loading more than 300 copies of a molecule’s DNA nanospheres into a patterned nanoarray. BGISEQ-500 system (BGI-Shenzhen, China) was used to sequence the cDNA library, and the read length was 100 bp.

Clean reads were obtained by screening the sequencing data, subsequently using Bowtie2 to map them to the genome of the watermelon in CuGenDB (http://cucurbitgenomics.org/organism/21, 13 January 2022). FPKM (fragments per kilobase million) was used to calculate gene expression levels. Gene annotation and function assignment were performed based on KEGG (http://www.genome.jp/kegg/, 27 February 2022) and GO (http://www.geneontology.org/, 27 February 2022) databases. DEGswere set as the following: gene fold change ≥ 2.00 and FDR (false discovery rate) ≤ 0.001. Through the GO enrichment and KEGG enrichment pathway, the metabolic pathways with significant enrichment were identified and compared with the genome-wide background. According to GO and KEGG annotation results and official classification, the DEGs were functionally classified. Under normal circumstances, FDR ≤ 0.01 is considered as significant enrichment.

### 2.4. RT-qPCR for DEGs

Total RNA was extracted using the Plant RNA Kit (Huayueyang Biotechnology Co., Ltd., Beijing, China). A total of 1.0 μg RNA was used for synthesizing cDNA by using the PrimeScript RT reagent Kit with gDNA Eraser (TaKaRa) according to the manufacturer’s protocol. All primers were synthesized by SunYa (Zhengzhou, China). Quantitative real-time PCR was performed on the Light Cycler480 Real-Time System (Bio-Rad Laboratories) in the following steps: 45 cycles of 95 °C for 5 min, 95 °C for 10 s, 58 °C for 10 s, and 72 °C for 10 s, followed by a melting curve analysis. Each reaction mixture (final volume: 20 μL) contained 1.0 μL previously diluted cDNA (1:5). All primers are shown in Supplementary Table S1. *Actin* was used as the reference gene, and the primer sequences used were as follows: forward primer: 5′-GAACTTGGCACCTGTCCTGT-3′ and reverse primer: 5′-GAACAGTGCAACAGCCTCAA-3′. Relative gene expression values were calculated using the 2^−ΔΔCt^ method [15].

## 3. Results

### 3.1. Salt Stress Inhibits the Growth of Watermelon Seedlings

**Figure 1:**
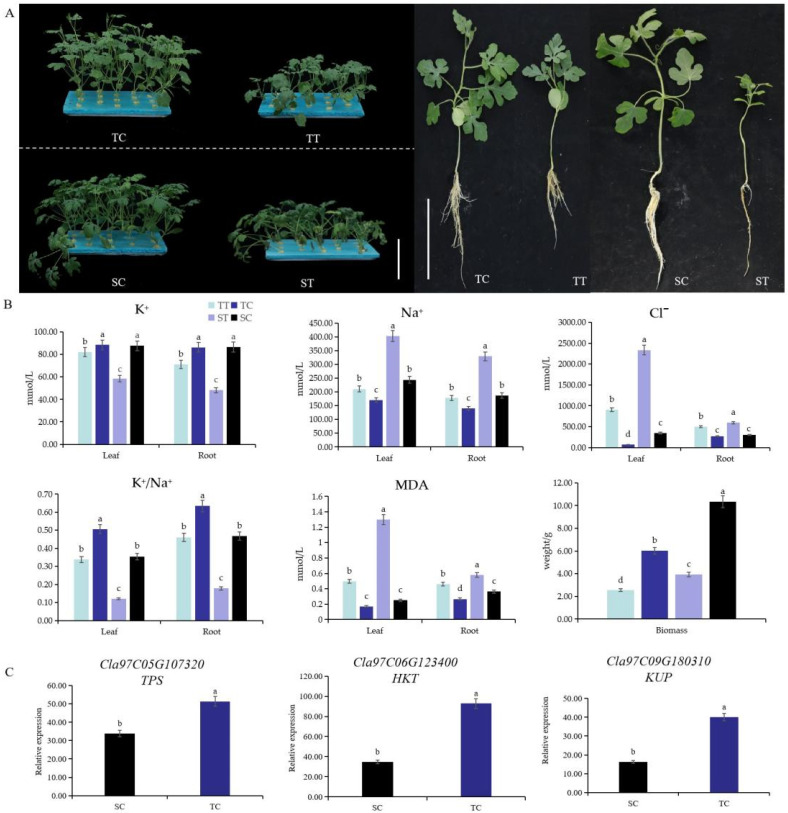
The influences of 150 mmol·L^−1^ NaCl treatment on the growth of watermelon seedlings. (**A**) Phenotype of three-leaves period of salt-tolerant variety and salt-sensitive variety seedlings in Hoagland solution for two weeks. Scale bar is 5 cm. (**B**) Measurements of the K^+^, Na^+^ Cl^−^, MDA content, REC Na^+^ content, and Cl^−^ content in leaves and roots. Error bars indicate SE (*n* = 3). (**C**) Expression levels of genes related to salt tolerance in roots. Little letters indicate significant differences among the four treatments (*p* < 0.05). TT represents salt-tolerant variety under NaCl treatment, TC represents salt-tolerant variety under control, ST represents salt-sensitive variety under NaCl treatment, SC represents salt-sensitive variety under control.

Under 150 mmol·L^−1^ NaCl stress, the growth of the salt-tolerant variety and the salt-sensitive variety watermelon seedlings was significantly inhibited, and the height of the plant was significantly lower than that of the control (Figure 1A). For the two varieties, the growth of the salt-tolerant variety was better than that of the salt-sensitive variety; for example, leaves of the salt-tolerant variety grew normally under 150 mmol·L^−1^ NaCl treatment, while the leaves of the salt-sensitive variety withered or even died.

We further measured physiological indexes to analyze the difference between the two varieties under salt stress, including MDA content, Na^+^ content, Cl^−^ content, and K^+^ content (Figure 1B). For the four indexes, the value of the two varieties significantly increased after NaCl treatment both in leaves and roots, and the variation range of the salt-sensitive variety was higher than that of the salt-tolerant variety. Moreover, the expression levels of genes *Cla97C05G107320* (trehalose-6-phosphate synthase, *TPS*), *Cla97C06G123400* (high-affinity K^+^ transporter, *HKT*), and *Cla97C09G180310* (K^+^ uptake permease, *KUP*) related to salt tolerance in salt-tolerant variety roots were significantly higher than that of salt-sensitive variety roots (Figure 1C). All the results suggested that salt-tolerant varieties absorbed less Na^+^ and Cl^−^, absorbed more K^+^, and suffered much less damage than the salt-sensitive variety under 150 mmol·L^−1^ salt stress.

### 3.2. RNA-Seq for the Roots and Leaves of Watermelon under Salt Treatment

In order to analyze the related genes of watermelon response to salt stress, transcriptome sequencing was performed on watermelon seedling samples of the salt-tolerant variety and the salt-sensitive variety before and after 150 mmol·L^−1^ NaCl stress, including leaves and roots, with three biological replicates for each group, with a total of twenty-four samples. After data filtering, each sample produced an average of 6.33 Gb of data, of which data quality Q30 was greater than 91%, the average genome alignment rate was 93.26%, and the average gene set alignment rate was 66.65% (Supplementary Table S2). In general, the transcriptome data were of reliable quality and could be used for subsequent analysis.

**Figure 2:**
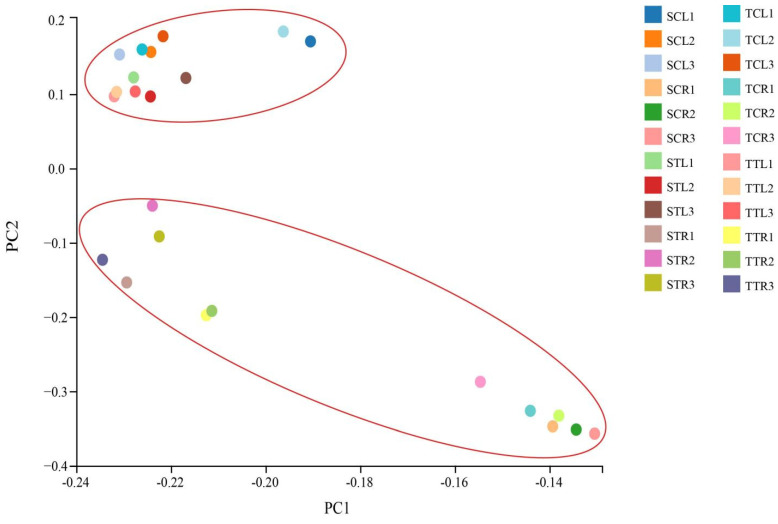
PCA of the 24 samples based on the FPKM. The X and Y axis represent the new data set of corresponding principal components obtained after dimension reduction of FPKM values. Each dot represents each sample. TT represents salt-tolerant variety under NaCl treatment, TC represents salt-tolerant variety under control, ST represents salt-sensitive variety under NaCl treatment, SC represents salt-sensitive variety under control. R represents root, and L represents leaf.

A total of 21,069 genes were detected in all samples, including 20,432 known genes and 637 predicted genes, 1412 of which encoding transcription factors. Then, the correlation among the different samples was analyzed based on principal component analysis (PCA). PCA maps showed that the root and leaf samples were clearly divided into two groups (Figure 2), indicating that the gene expression profiles of these samples were reliable.

**Figure 3:**
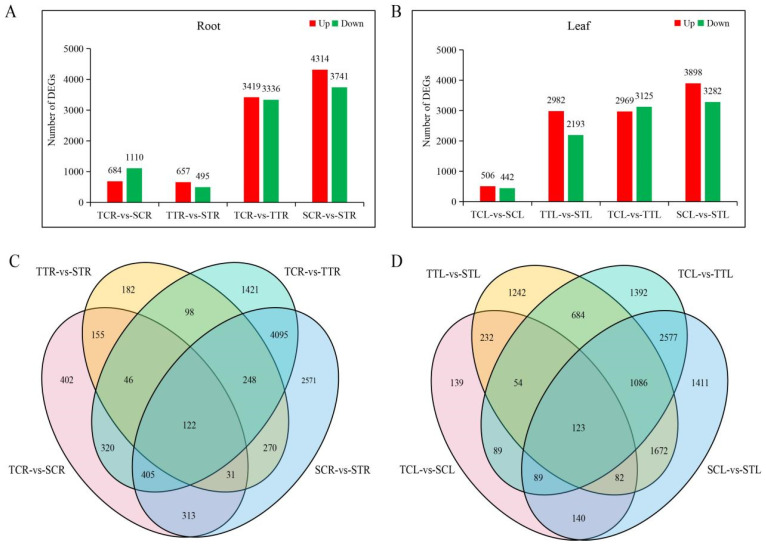
Statistics for DEGs under 150 mmol·L^−1^ NaCl treatment in comparison groups. (**A**) Number of up- and down-regulated DEGs in the root. (**B**) Number of up- and down-regulated DEGs in the leaf. (**C**) Venn diagrams of DEGs for comparison groups in the root. (**D**) Venn diagrams of DEGs for comparison groups in the leaf. TT represents salt-tolerant variety under NaCl treatment, TC represents salt-tolerant variety under control, ST represents salt-sensitive variety under NaCl treatment, SC represents salt-sensitive variety under control. R represents root and L represents leaf.

In roots, the number of DEGs before salt treatment (TCR-*vs*-SCR, 1794) was significantly higher than that after salt treatment (TTR-*vs*-STR, 1152), and the number of DEGs in salt-sensitive variety before and after salt treatment (SCR-*vs*-STR, 8055) was significantly higher than that in salt-tolerant variety (TCR-*vs*-TTR, 6755) (Figure 3A). In leaves, the number of DEGs before salt treatment (TCL-*vs*-SCL, 948) was significantly lower than that after salt treatment (TTL-*vs*-STL, 5175), and the number of DEGs in the salt-sensitive variety before and after salt treatment (SCL-*vs*-STL, 7180) was also significantly higher than that in salt-tolerant variety (TCL-*vs*-TTL, 6094) (Figure 3B). These results indicated that watermelon responded to salt stress through a series of gene changes, and the number of DEGs in the salt-sensitive variety was significantly higher than that in the salt-tolerant variety, which might be due to more severe damage under salt stress. In addition, there were 10,679 DEGs in roots and 122 shared DEGs in the four comparison groups (TCR-*vs*-SCR, TTR-*vs*-STR, TCR-*vs*-TTR and SCR-*vs*-STR) (Figure 3C); there were 10,679 DEGs in leaves and 123 shared DEGs in the four comparison groups (TCL-*vs*-SCL, TTL-*vs*-STL, TCL-*vs*-TTL, and SCL-*vs*-STL) (Figure 3D) (Supplementary Table S3), suggesting that these genes might play important roles in watermelon response to salt stress.

### 3.3. Verification of DEGs by RT-qPCR

**Figure 4:**
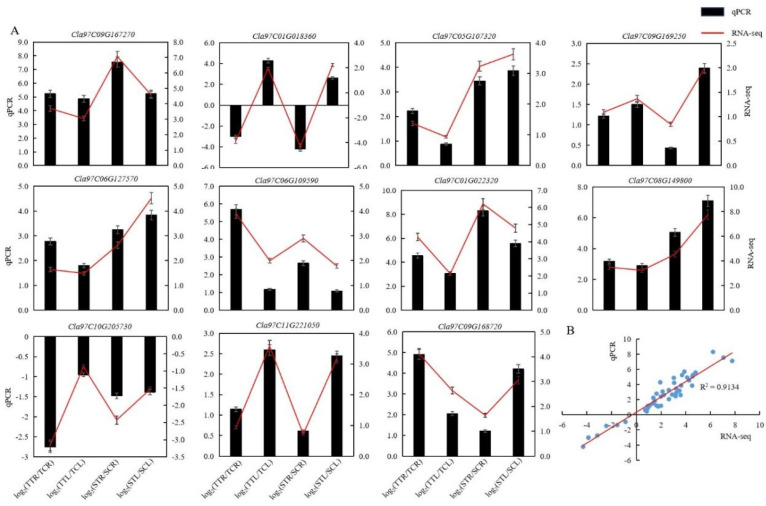
Verification of DEGs by RT-qPCR. (**A**) Expression patterns of DEGs of RT-qPCR and RNA-aeq data. (**B**) Correlation analysis of RT-qPCR and RNA-seq data. The value in each graph represents fold change.

To verify the accuracy of RNA-seq results and examine the expression patterns of DEGs, 11 DEGs (*Cla97C09G167270*, *Cla97C01G018360*, *Cla97C05G107320*, *Cla97C09G169250*, *Cla97C06G127570*, *Cla97C06G109590*, *Cla97C01G022320*, *Cla97C08G149800*, *Cla97C10G205730*, *Cla97C11G221050*, and *Cla97C09G168720*) were randomly selected to conduct qPCR (Figure 4A). The results showed that the expression patterns of 11 DEGs were highly consistent with those of genes in RNA-seq data. In addition, the correlation analysis showed that the determination coefficient (R^2^) of RT-qPCR and RNA-seq data was 0.9134 (Figure 4B), which demonstrated that the RNA-seq data are reliable and can be used for further analysis.

### 3.4. GO Classification and KEGG Pathway Enrichment Analysis of DEGs

**Figure 5:**
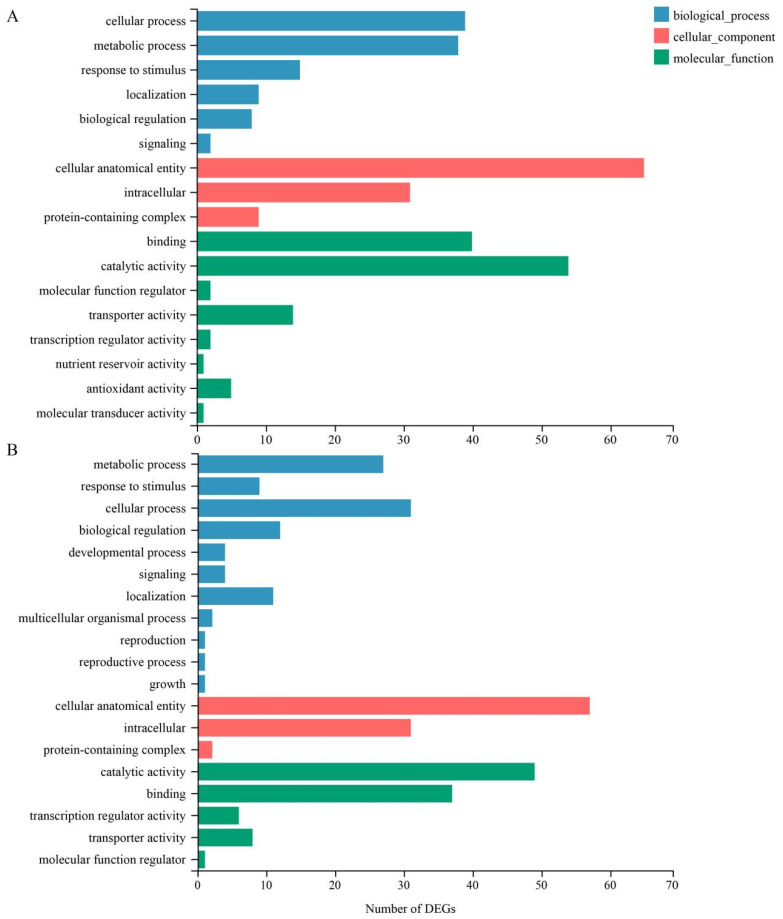
GO classification of DEGs. (**A**) GO Classification of DEGs in the root. (**B**) GO Classification of DEGs in the leaf.

In order to analyze the main response pathways of watermelon seedlings to salt stress, the shared DEGs of the root and leaf were mapped to the GO database, respectively. All DEGs were classified on the basis of biological process, cell component, and molecular function (Figure 5). In the roots, 122 DEGs were classified into 17 GO terms, including 6 terms of biological process, among which cellular process (39) and metabolic process (38) contained the most genes; 3 terms of cell component, among which cellular element contained the most genes (65); 8 terms of molecular function, of which catalytic activity (54) and binding (40) contained the most genes (Figure 5A). In leaves, 123 DEGs were classified into 19 GO terms, including 11 terms of biological process, among which cellular process (31) and metabolic process (27) contained the most genes; three terms of cell component, among which cellular element (57) contained the most genes; 5 terms of molecular function, with catalytic activity (49) and binding (37) containing the most genes (Figure 5B). By further analysis, we found that there were 14 shared GO terms in the root and leaf, accounting for 82.4% and 72.7% of the total number of GO terms in the root and leaf, respectively. Taken together, these results indicated that a large number of genes that relate to biological process, cell component, and molecular function are activated after salt stress treatment.

**Figure 6:**
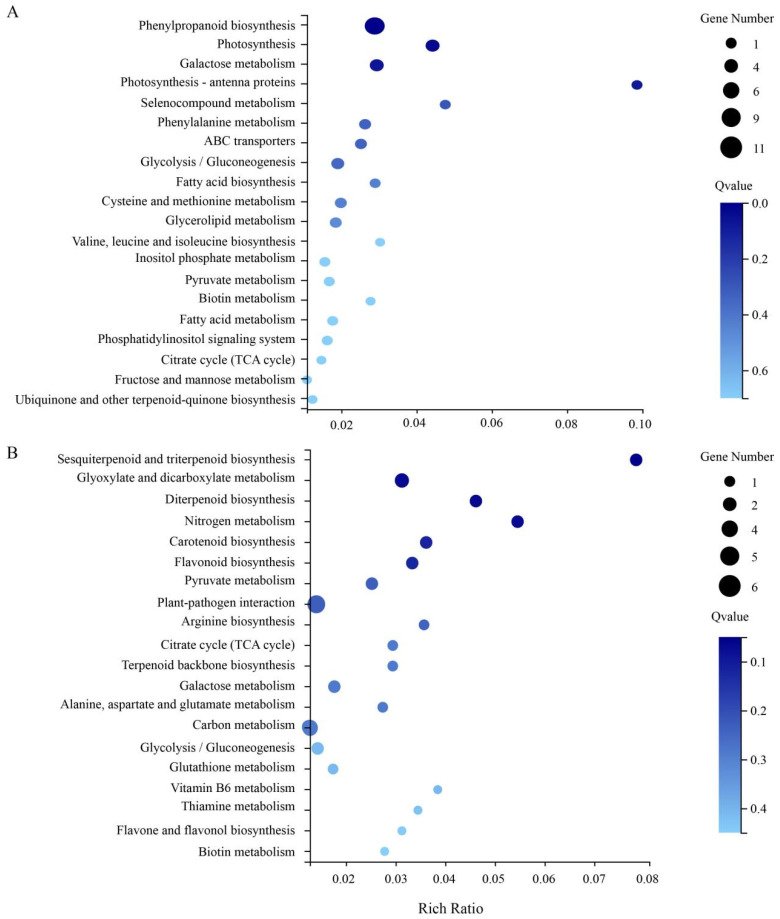
KEGG pathway enrichment analysis of DEGs. (**A**) KEGG pathway enrichment analysis of DEGs in the root. (**B**) KEGG pathway enrichment analysis of DEGs in the leaf.

Furthermore, we performed KEGG enrichment to analyze the functions of shared DEGs of the root and leaf from metabolic pathways, and 20 top enriched metabolic pathways were selected (Figure 6). In roots, the main enriched pathways were phenylpropanoid biosynthesis, photosynthesis, galactose metabolism, photosynthesis-antenna proteins, pyruvate metabolism, citrate cycle (TCA cycle), glycolysis/gluconeogenesis, and biotin metabolism, etc., and phenylpropanoid biosynthesis pathway contained the most genes (9) (Figure 6A). In leaves, the main enriched pathways were plant-pathogen interaction, glyoxylate and dicarboxylate metabolism, sesquiterpenoid and triterpenoid biosynthesis, pyruvate metabolism, citrate cycle (TCA cycle), galactose metabolism, glycolysis/gluconeogenesis, and biotin metabolism, etc. (Figure 6B). Interestingly, we found that there were five shared pathways in the root and leaf KEGG pathway enrichment, including pyruvate metabolism (ko00620), citrate cycle (TCA cycle, ko00020), galactose metabolism (ko00052), glycolysis/gluconeogenesis (ko00010), and biotin metabolism (ko00780), and most of the genes contained in the pathways were related to sugar or energy (ATP or NADP^+^/NADPH) (Table 1). These results suggest that the metabolic process of watermelon seedlings under salt stress is complex and induces the expression of ATP- or NADPH-related genes.

### 3.5. Transcription Factors Respond to Salt Stress

**Figure 7:**
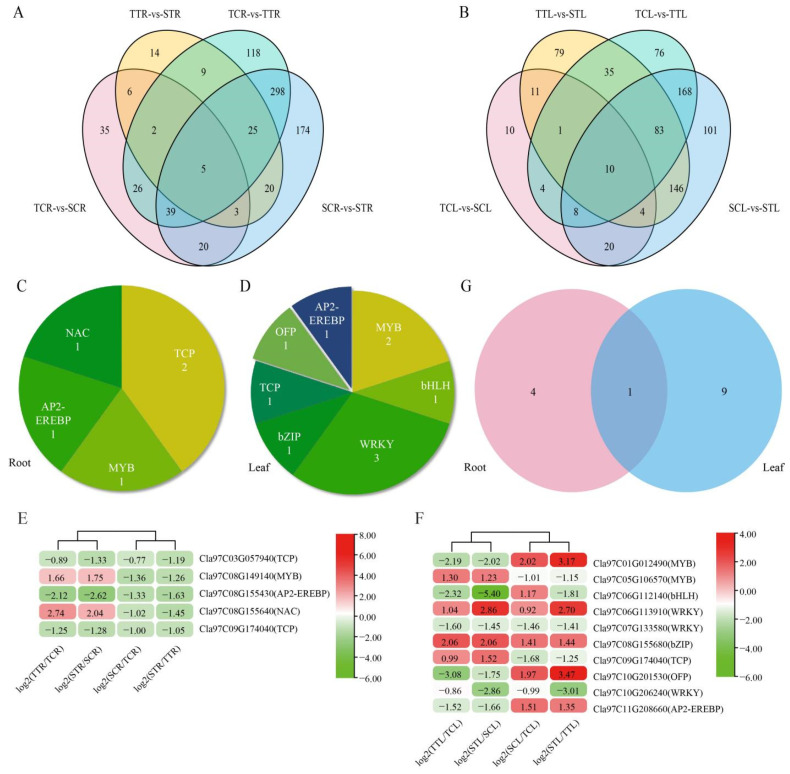
Analysis of differentially expressed TFs in the roots and leaves under 150 mmol·L^−1^ NaCl treatment. (**A**) Venn diagrams of TFs in the root. (**B**) Venn diagrams of TFs in the leaf. (**C**) The shared TFs of four comparison groups in the root. (**D**) The shared TFs of four comparison groups in the leaf. Expression levels of genes that encode TFs in the root (**E**) and leaf (**F**). (**G**) Venn diagrams of shared TFs in the root and leaf. TT represents salt-tolerant variety under NaCl treatment, TC represents salt-tolerant variety under control, ST represents salt-sensitive variety under NaCl treatment, SC represents salt-sensitive variety under control. R represents root and L represents leaf.

Transcription factors regulate various biological processes by directly targeting downstream genes. Many transcription factors were identified from DEGs in the root and leaf under salt stress (Figure 7). In roots, 79 transcription factors were found to be differentially expressed in the four comparison groups, among which 5 were shared (Figure 7A). In leaves, 88 transcription factors were found to be differentially expressed in the four comparison groups, of which 10 were shared (Figure 7B). We counted the subfamilies of these shared transcription factors according to the annotations of each gene (Figure 7C,D). Results showed that the five transcription factors in roots were divided into four subfamilies including 1 NAC (Cla97C08G155640), 1 AP2-EREBP (Cla97C08G155430), 1 MYB (Cla97C08G149140), and 2 TCP (Cla97C03G057940, Cla97C09G174040) (Figure 7C); among them, the expression levels of *Cla97C08G149140* and *Cla97C08G155640* were significantly up-regulated by salt stress and were significantly up-regulated in the salt-tolerant variety (Figure 7E). Ten transcription factors in leaves were divided into seven subfamilies including 1 AP2-EREBP (Cla97C11G208660), 1 OFP (Cla97C10G201530), 1 TCP (Cla97C09G174040), 1 bZIP (Cla97C08G155680), 1 bHLH (Cla97C06G112140), 2 MYB (Cla97C01G012490, Cla97C05G106570), and 3 WRKY (Cla97C06G113910, Cla97C07G133580, Cla97C10G206240) (Figure 7D); among them, the expression levels of *Cla97C05G106570*, *Cla97C09G174040*, *Cla97C06G113910*, and *Cla97C08G155680* were significantly up-regulated by salt stress, of which Cla97C05G106570 and Cla97C09G174040 were significantly up-regulated in the salt-tolerant variety (Figure 7F). It was worth noting that in these subfamilies, one TCP transcription factor (Cla97C09G174040) was shared in roots and leaves (Figure 7G), indicating that TCP transcription factor significantly responds to salt stress. These results suggested that transcription factors, especially TCP, play a crucial role in the responses to salt stress of watermelon.

### 3.6. DEGs in Response to Salt Stress

**Figure 8:**
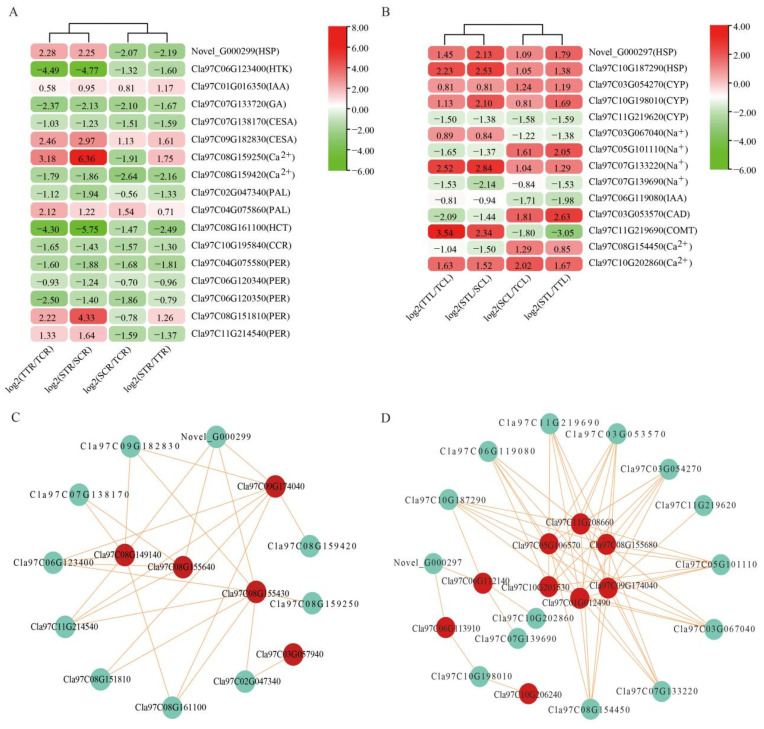
DEGs respond to salt stress in watermelon seedlings. (**A**) Heat map of DEGs in response to salt stress in the root. (**B**) Heat map of DEGs in response to salt stress in the leaf. (**C**) Co-expression network analysis of transcription factors and DEGs in response to salt stress in the root. (**D**) Co-expression network analysis of transcription factors and DEGs in response to salt stress in the leaf. The red dots represent transcription factors, and the turquoise dots represent genes. TT represents salt-tolerant variety under NaCl treatment, TC represents salt-tolerant variety under control, ST represents salt-sensitive variety under NaCl treatment, SC represents salt-sensitive variety under control. R represents root and L represents leaf.

In order to comprehensively understand the expression of salt-tolerant genes in watermelon under salt stress, we screened DEGs in roots and leaves, mainly including genes related to heat shock protein (HSP), cytochrome P450 (CYP), potassium, sodium, calcium, cell wall, lignin, and plant hormone (Figure 8). In roots, one HSP (Novel_G000299), one potassium-related gene (Cla97C06G123400), two calcium-related genes (Cla97C08G159250, Cla97C08G159420), two plant hormone-related genes (Cla97C01G016350, Cla97C01G016350), two cell wall-related genes (Cla97C07G138170, Cla97C09G182830), nine lignin-related genes (Cla97C02G047340, Cla97C04G075860, Cla97C08G161100, Cla97C10G195840, Cla97C04G075580, Cla97C06G120340, Cla97C06G120350, Cla97C08G151810, and Cla97C11G214540), of which Cla97C11G214540, Cla97C01G016350, Cla97C09G182830, Cla97C08G159250, Cla97C04G075860, Cla97C08G151810, and Cla97C11G214540 were significantly up-regulated, were induced by salt stress (Figure 8A). In leaves, two HSPs (Novel_G000297, Cla97C10G187290), three CYP-related genes (Cla97C03G054270, Cla97C10G198010, Cla97C11G219620), four sodium-related genes (Cla97C03G067040, Cla97C05G101110, Cla97C07G133220, Cla97C07G139690), two calcium-related genes (Cla97C08G154450, Cla97C10G202860), one plant hormone-related gene (Cla97C06G119080), and two lignin-related genes (Cla97C03G053570, Cla97C11G219690), of which Novel_G000297, Cla97C10G187290, Cla97C03G054270, Cla97C10G198010, Cla97C03G067040, Cla97C07G133220, Cla97C11G219690, and Cla97C10G202860 were significantly up-regulated, were induced by salt stress (Figure 8B). 

Then, we performed the co-expression network to explore the correlation between transcription factors and salt-tolerant related genes, and the selected correlation coefficient was all greater than 0.9. In roots, the co-expression network contained five transcription factors and 10 DEGs, including one HSP (Novel_G000299), one potassium-related gene (Cla97C06G123400), two cell wall-related genes (Cla97C07G138170, Cla97C09G182830), two calcium-related genes (Cla97C08G159250, Cla97C08G159420), and four lignin-related genes (Cla97C02G047340, Cla97C08G151810, Cla97C08G161100, Cla97C11G214540), resulting in 26 network lines (Figure 8C), and TCP (Cla97C09G174040, r = 0.997) and MYB (Cla97C08G149140, r = −0.998) transcription factor had the highest correlation coefficient with potassium-related genes (Supplementary Table S4).

In leaves, the co-expression network contained 10 transcription factors and 26 DEGs, including two HSPs (Novel_G000297, Cla97C10G187290), three CYP-related genes (Cla97C03G054270, Cla97C10G198010, Cla97C11G219620), two calcium-related genes (Cla97C08G154450, Cla97C10G202860), two lignin-related genes (Cla97C03G053570, Cla97C11G219690), one plant hormone-related gene (Cla97C06G119080), four sodium-related genes (Cla97C03G067040, Cla97C05G101110, Cla97C07G133220, Cla97C07G139690), resulting in 59 network lines (Figure 8D), and TCP (Cla97C09G174040, r = 1.000) and MYB (Cla97C05G106570, r = −1.000) transcription factor had the highest correlation coefficient with sodium-related genes (Supplementary Table S5). These results suggested that the response of watermelon to salt stress is realized through a complex gene regulatory network, and TCP and MYB transcription factors may play an important role in salt tolerance of watermelon.

### 3.7. Key DEGs Related to Salt Stress in Watermelon

**Figure 9:**
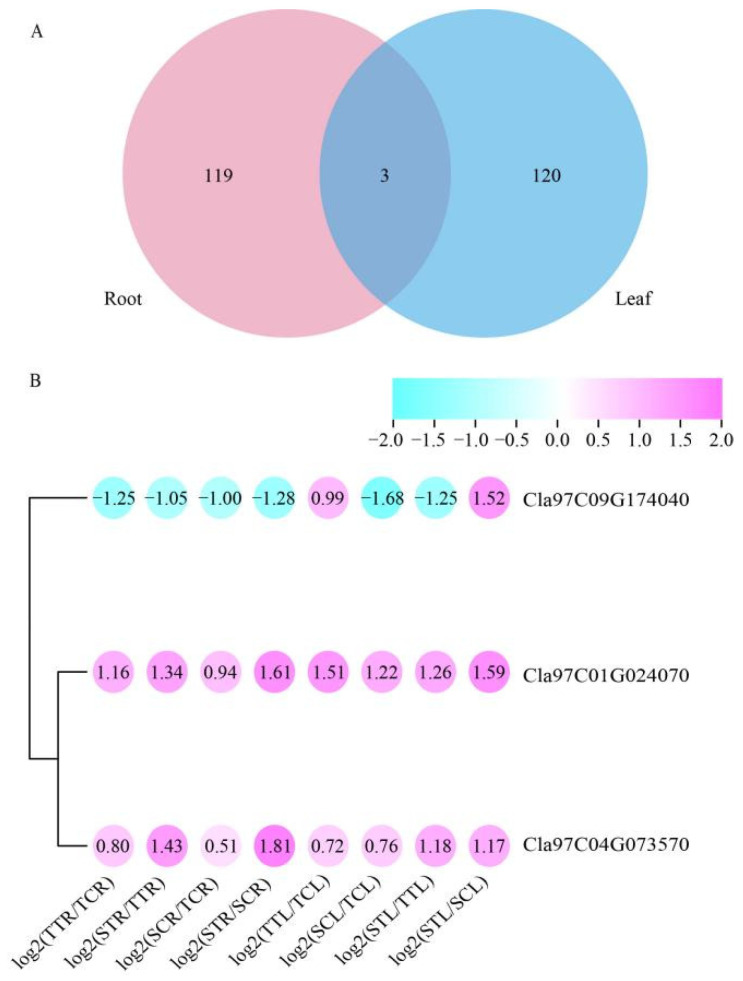
Screen of key DEGs response to salt stress in watermelon seedlings. (**A**) Venn diagrams of shared DEGs of the root and leaf. (**B**) Heat map of shared DEGs of shared DEGs of the root and leaf.

In order to further identify the key genes in response to salt stress in watermelon, we analyzed the shared DEGs through all the comparison groups (Figure 9). The results showed that there were only three shared DEGs between the root and leaf (Figure 9A), including one TCP transcription factor (Cla97C09G174040), one bromodomain-containing protein (Cla97C01G024070), and one SCO1-like protein (Cla97C04G073570). Among the three DEGs, both *Cla97C01G024070* and *Cla97C04G073570* were up-regulated in all eight comparison groups, indicating that they were induced by salt stress, but the expression levels of the salt-tolerant variety were lower than that of the salt-sensitive variety; however, for *Cla97C09G174040*, it was significantly up-regulated by salt stress in the leaf, and the expression levels of the salt-tolerant variety were higher than that of salt-sensitive variety both in the root and leaf (Figure 9B), suggesting that TCP transcription factors play a crucial role in salt tolerance of watermelon.

## 4. Discussion

Biomass is a key indicator for plant growth under salt stress [6]. Overall, salt stress reduces plant biomass, but the difference in the degree of decline was determined by species. Biomass of tomato [16] and sunflower [17] decreased significantly under 50 mmol·L^−1^ NaCl treatment, while the biomass of wheat, rice, and maize decreased significantly under 100–150 mmol·L^−1^ NaCl treatment [1]. However, biomass of halophyte *Salicornia europaea* began to decrease when NaCl concentration was higher than 400 mmol·L^−1^ [18]. In addition, Han [12] found that biomass, growth potential, and photosynthesis of watermelon seedlings decreased significantly under salt stress. In this study, the biomass of salt-tolerant and salt-sensitive watermelon seedlings were both significantly reduced, and the decrease degree of salt-tolerant watermelon varieties was less than that of salt-sensitive watermelon varieties, which decreased by 43.0% and 47.7%, respectively (Figure 1); the results were similar to previous studies. Na^+^ or Cl^−^ concentration, K^+^ content, and Na^+^/K^+^ ratio in leaves and roots under salt stress are often used to reflect the degree of salt stress in plants [6]. Han et al. [19] proved that Na^+^ accumulated in the roots and stems of watermelon seedlings, Cl^−^ accumulated in roots, and K^+^ content decreased significantly in stems. Guo et al. [20] found that wild watermelon had strong salt tolerance, possibly because its root had a stronger ability to intercept Na^+^ and transport K^+^. In this study, salt stress significantly reduced K^+^ content and K^+^/Na^+^ ratio in roots and leaves, and increased Na^+^ and Cl^−^ accumulation. We also found that Cl^−^ and Na^+^ accumulated more in leaves, and K^+^ accumulated in roots (Figure 1).

MDA is one of the main products of peroxidation of membrane-lipid, and also an important indicator to reflect the degree of plasma membrane damage. MDA content is an important index reflecting the damage degree of plasma membrane. Previous studies showed that the content of MDA in Carex and soybean increased significantly after salt stress treatment [21,22]. In addition, studies showed that with the increase of salt concentration, the cell membrane structure of watermelon seedlings was damaged, the MDA content and relative permeability of the plasma membrane were significantly increased, the metabolism of ROS was disordered, and the $O_{2}^{-.}$ production rate of seedling leaves was significantly increased [21,23]. In our data, the MDA content also increased significantly after salt stress treatment both in leaves and roots, and the increased degree of salt-tolerant watermelon varieties was less than that of salt-sensitive watermelon varieties (Figure 1).

Under salt stress, various plant hormones respond to salt stress. For example, the reduction of plant growth and development under stress may be the result of changes in plant auxin accumulation and redistribution [24]. The primary expression genes induced by auxin were *GH3* (*gretchen hagen 3*), *Aux/IAA*, and *SAUR* (*small auxin up RNA*) [25]. *GH3* family encodes a class of enzymes that catalyze the coupling of auxin and amino acids, which can regulate plant growth and development to be adapted to external changes. *GH3* can interact with ARF (auxin response factor) to induce the expression of downstream genes [26]. In this study, the expression of *GH3* (*Cla97C01G016350*) increased in roots, which ensured the growth of watermelon seedlings under salt stress. Furthermore, *AUX/IAA* played a central role in the regulation of auxin response and inhibited the expression of auxin-related genes by binding to ARF as an inhibitor of auxin-induced genes [27]. However, *Cla97C06G119080* (*IAA16*) was down-regulated, which indicated that it maintains the growth of watermelon seedlings under salt stress. Under high salt stress, the gibberellins (GA_1_ and GA_4_) content decreased, and the DELLA protein content increased, which enhanced tolerance to salt stress by inhibiting plant growth [28]. In our study, one GA_4_-related gene (*Cla97C07G133720*) was significantly down-regulated under salt stress, which may be the reason for the inhibition of watermelon seedling growth and adaptation to salt stress. Furthermore, GA can stimulate the H^+^–ATPase activity of the vacuole membrane and form the electrochemical gradient of H^+^ across the vacuole membrane, which provides a driving force for the secondary active transport of various solute molecules (anions, cations, amino acids, and carbohydrates) across the vacuole membrane and increases the germination rate of seeds under salt stress [29]. In addition, the Na^+^/H^+^ reverse transporter on the plasma membrane can use ATP on the plasma membrane to expel Na^+^ from the cells. The sodium hydrogen pump on the vacuole membrane uses the energy provided by ATP on the vacuole membrane to compartmentalize the Na^+^ region into the vacuole, thereby reducing the impact of excessive Na^+^ accumulation on the cytoplasm [30]. In wheat, the high-density thylakoids of wheat under salt stress may produce more ATP and NADPH, thereby increasing the energy supply and improving the response rate of wheat to salt stress [31]. It was worth noting that many KEGG pathways enriched by DEGs in roots and leaves are related to energy metabolism (Table 1). For example, we screened two genes that encode alcohol dehydrogenase; one (*Cla97C02G031490*) of them was down-regulated and another (*Cla97C02G031490*) was up-regulated under salt stress. A previous study proved that overexpression of *Sysr1* (a member of the alcohol dehydrogenase superfamily) in tobacco induced stress-related gene expression and increased tolerance to salt stress [32]. In this study, there was one down-regulated gene (*Cla97C05G107020*) that encodes pyruvate dehydrogenase E1 under NaCl stress, and in *A.thaliana*, mutation of *IAR4* (a member of the pyruvate dehydrogenase E1 superfamily) led to an accumulation of greater Na^+^ and exhibited a greater Na^+^/K^+^ ratio under NaCl treatment by reducing *SOS1* and *SOS3* expression, resulting in increasing sensitivity to salt stress [33]. We also found two genes (*Cla97C10G192070* and *Cla97C11G220850*) that encode phosphoenolpyruvate carboxykinase (PEPCK), and both of them were up-regulated under NaCl stress. In rice, the expression of *PEPCK* was down-regulated in early stages and tended to respond to salt stress during maturity under NaCl stress [34]. These results indicated that energy might be required in the Na^+^ efflux process, and watermelon seedlings respond to salt stress by changing the expression of these genes.

The cell wall is the primary line of defense for plants to resist salt stress [35]. When plant roots are subjected to external salt stress, the cell wall sensing system takes the lead to receive salt stress signals [36,37]. Cellulose is the main component of the cell wall, which is synthesized by cellulose synthases (CESAs) guided by microtubules on the plasma membrane [38]. A previous study found that the content of plant cellulose decreased under salt stress [39]. In roots, we found two genes encoding CESA, of which *Cla97C07G138170* was down-regulated under salt stress, whereas *Cla97C09G182830* was up-regulated, suggesting that these *CESAs* have different expression patterns in response to salt stress. Many ion transporters related to salt tolerance have been discovered in recent twenty years. Among them, HKT plays a key role in K^+^/Na^+^ absorption, long-distance transport, and redistribution [40,41]. Research on barley showed that Na^+^ accumulated in roots and leaves when *HvHKT1:1* was knocked out, while overexpression of *HvHKT1:1* in salt-sensitive *Arabidopsis* mutant *hkt1-4* and *sos1-12* caused Na^+^ content to decrease significantly in roots and shoots, indicating that *HvHKT1:1* plays an important role in Na^+^ transport in roots [42]. Recent studies have found that HvHKT1 significantly reduced Na^+^ transport from roots to shoots and increased K^+^/Na^+^ ratio compared with wild type [43]. Kader et al. [44] found that *OsHKT1* and *OsHKT2* were induced under salt stress, and Na^+^ was reduced by regulating the Na^+^/K^+^ ratio. In this study, we found one *HTK* (*Cla97C06G123400*) in roots, which was down-regulated under salt stress, but its expression in the salt-tolerant variety was significantly higher than that in salt-sensitive variety, indicating that *HTK* plays a key role in Na^+^ transport in roots of salt-tolerant variety. CYP450s are a class of monooxygenases encoded by the supergene family, which mainly exist in animals and plants and microorganisms, and play an important role in biological defense [45]. For example, secondary metabolites produced by amino acids catalyzed by CYP71A are related to plant defense and stress resistance [46]. In leaves, there were three genes encoding *CYP450* (*Cla97C03G054270*, *Cla97C10G198010*, and *Cla97C11G219620*), among which *Cla97C03G054270* belonged to *CYP71A1* and was markedly up-regulated under salt stress. Lignin is the second most abundant biopolymer in plants, which mainly exists in the secondary wall of vessel molecules [47]. Under salt stress, the degree of lignification of the plant root cell wall will increase, which can not only effectively prevent the intracellular ion absorption, but also enhance the structural rigidity and firmness of the conduction tissue and improve the salt tolerance of plants. Salt stress can change many enzymes involved in lignin biosynthesis, generally by changing their expression patterns, thereby regulating lignin synthesis and responding to salt stress [48]. For example, the expression of tomato peroxidase gene *TPX1* was up-regulated under salt treatment, and the lignin content in transgenic tomato leaves with high expression of *TPX1* increased, indicating that *TPX1* may be involved in the lignification of root and aboveground parts [49]. In roots, we found nine lignin biosynthesis-related DEGs, five of which were peroxidase genes (*PERs*), suggesting that they were involved in the response of watermelon seedlings to salt stress.

Under salt stress, transcription factors regulate their expression levels by changing the expression of different genes, such as bZIP, WRKY, AP2/ERF, MYB, bHLH, NAC, etc. In soybean, overexpression of *GmbZIP2* in hairy roots enhanced the expression of stress response genes *GmMYB48*, *GmWD40*, *GmDHN15*, *GmGST1*, and *GmLEA* [50]. Transcription factors *bHLH* and *WRKY* enhanced salt tolerance of *Arabidopsis* by regulating the expression of salt response gene *AtKUP2* [51]. *AtMYB20* in *Arabidopsis* influenced plant resistance to salt stress by regulating the ABA signaling pathway. Salt stress and ABA stress induced *AtMYB20* gene expression. Overexpression of *AtMYB20* gene can enhance the resistance of transgenic *Arabidopsis* to salt stress and reduce the expression levels of ABA signal negative regulatory genes *ABI1*, *ABI2,* and *AtPP2CA* under salt stress [52]. In addition, overexpression of *SlMYB102* in tomato increased the K^+^/Na^+^ ratio and the activity of active oxygen scavenging enzyme, as well as the transcription abundance of salt stress-related genes in the two overexpression lines was up-regulated [53]. Ju et al. [54] reported that overexpression of *VvNAC17* in *Arabidopsis* enhanced drought and salt tolerance and up-regulated the expression of ABA and stress-related genes *ABI5*, *AREB1*, *COR15A*, *COR47,* and *P5CS*. In roots and leaves, we screened five and ten differentially expressed TFs, respectively (Figure 7C), and found a strong correlation between them and salt tolerance-related genes, most of which were greater than 0.9 (Figure 8C,D), indicating that these TFs may improve salt tolerance of watermelon seedlings by regulating the expression of corresponding genes. Among the TFs, TCP (Cla97C09G174040, TCP10, Supplementary Figure S2) was most noteworthy, which was found in roots and leaves. Although it had different expression patterns in roots and leaves, its expression levels in the salt-tolerant variety were significantly higher than those in the salt-sensitive variety, indicating that it plays a very important role in the salt tolerance of watermelon seedlings.

TCP is a plant-specific transcription factor, which belongs to bHLH TF. TCP activates or inhibits gene expression through its bHLH domain interacting with other proteins [1]. For example, TCP4 binds to the promoter region of functional genes and regulates their expression, thus playing a key role in multiple growth and development processes [55]. In *Phyllostachys edulis*, *PeTCP10* was induced by drought and ABA, and PeTCP10 directly targeted stress-/ABA-responsive gene *BT2* to improve drought resistance [56]. In *Betula platyphylla*, *BpTCP10* was involved in the response to salt stress, and the transgenic *Betula platyphylla* lines showed salt sensitivity after inhibiting the expression of *BpTCP10* [57]. Micro RNAs are non-coding single-stranded RNAs that widely exist in the biological world and regulate the expression of their target genes to change the growth and development process of plants. In *Arabidopsis*, microRNA319 (miR319) has been demonstrated to target *TCP* genes to influence their growth and development. MiR319-TCP4 bound to the *cis*-acting element of *YUCCA5*, directly activated its transcription and expression, and promotes cell elongation of *Arabidopsis* hypocotyl [1]. In *Agrostis stolonifem*, the target gene *AsPCF5*/6/8/14 in *miR319a* overexpression plants was down-regulated, and the drought resistance and salt tolerance of transgenic lines were enhanced [58]. Overexpression of *miR319* gene in *Solanum habrochaites* can also improve the drought resistance and high temperature resistance of tomato [59]. In addition, the overexpression of *miR319* in switchgrass can promote ethylene synthesis and enhance the ability to resist salt stress [60]. In watermelon, *ClTCP10* and *AtTCP10* have high homology, while *AtTCP10* is regulated by *miR319* [61], suggesting that *ClTCP10* may be the target gene of *miR319*, which has an extremely important regulatory effect on the development of watermelon, leading to improvement of the salt tolerance of watermelon.

## 5. Conclusions

**Figure 10:**
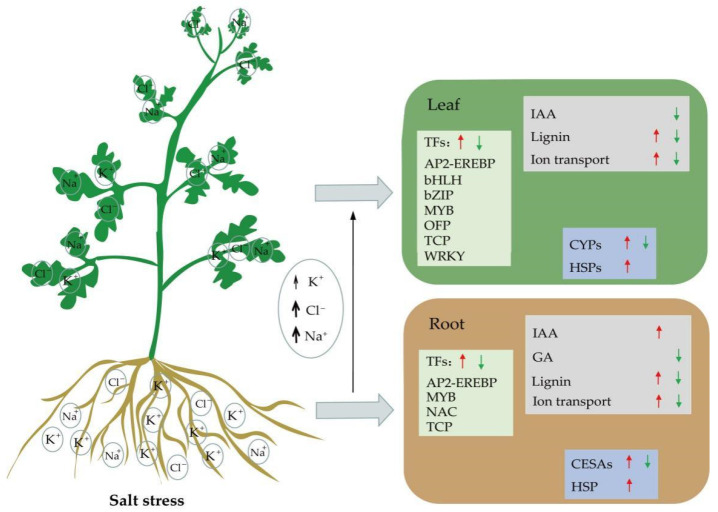
A putative schematic model of salt tolerance mechanism in watermelon seedlings. The ↑/↓ resprents the gene is up-regulated or down-regulated, respectively.

In conclusion, this study found that salt stress significantly reduced K^+^ content and K^+^/Na^+^ ratio in roots and leaves, and increased Na^+^, Cl^−^ accumulation, and MDA content in watermelon seedlings of salt-tolerant variety and salt-sensitive variety. Furthermore, based on our results, we proposed a hypothetical model describing the molecular mechanism of watermelon seedling response to salt stress (Figure 10). We suggest that the expression of some salt tolerance-related DEGs, such as plant hormone signal transduction pathway related genes, K^+^/Na^+^/Ca^2+^-related genes, and lignin biosynthesis-related genes, promoted the salt tolerance of watermelon. In addition, some DEGs related to sugar or energy pathways play a major role in ion transport. Moreover, transcription factors such as AP2-EREBP, bZIP, bHLH, MYB, NAC, OFP, TCP, and WRKY may also be associated with salt tolerance of watermelon by regulating above DEGs, especially a TCP TF (Cla97C09G174040).

## Figures and Tables

**Table 1:** Shared Genes involved in the KEGG pathway of the root and leaf.

Tissue	Gene ID	KEGG Number	Log2 (TT/TC)	Log2 (ST/SC)	Nr Description
Root	Cla97C03G054690	ko00620	1.85	6.31	malate synthase
	Cla97C05G107020	ko00620	−1.46	−1.25	pyruvate dehydrogenase E1
	Cla97C05G107020	ko00020	−1.46	−1.25	pyruvate dehydrogenase E1
	Novel_G000429	ko00052	−1.53	−2.74	UDP-sugar pyrophosphorylase
	Cla97C01G009200	ko00052	0.78	1.19	alpha-glucosidase
	Cla97C02G031490	ko00052	−2.47	−2.20	alcohol dehydrogenase (NADP^+^)
	Cla97C10G197800	ko00052	1.37	1.96	aldose 1-epimerase
	Cla97C10G197810	ko00052	1.37	1.96	alcohol dehydrogenase (NADP^+^)
	Cla97C02G031490	ko00010	−2.47	−2.20	alcohol dehydrogenase (NADP^+^)
	Cla97C05G107020	ko00010	−1.46	−1.25	pyruvate dehydrogenase E1
	Cla97C10G197800	ko00010	1.37	1.96	aldose 1-epimerase
	Cla97C10G197810	ko00010	1.37	1.96	aldose 1-epimerase
	Cla97C11G206680	ko00780	2.43	3.67	NADPH-dependent pterin aldehyde reductase-like
Leaf	Cla97C10G192070	ko00620	0.45	1.35	phosphoenolpyruvate carboxykinase [ATP]
	Cla97C11G216650	ko00620	−1.57	−1.64	lactoylglutathione lyase
	Cla97C11G220850	ko00620	1.00	3.73	phosphoenolpyruvate carboxykinase [ATP]
	Cla97C10G192070	ko00020	0.45	1.35	phosphoenolpyruvate carboxykinase [ATP]
	Cla97C11G220850	ko00020	1.00	3.73	phosphoenolpyruvate carboxykinase [ATP]
	Cla97C07G140230	ko00052	−0.58	−0.83	ATP-dependent 6-phosphofructokinase 6
	Cla97C08G156510	ko00052	−1.18	−1.42	beta-fructofuranosidase
	Cla97C08G156670	ko00052	−1.31	−1.47	beta-fructofuranosidase
	Cla97C07G140230	ko00010	−0.58	−0.83	ATP-dependent 6-phosphofructokinase 6
	Cla97C10G192070	ko00010	0.45	1.35	phosphoenolpyruvate carboxykinase [ATP]
	Cla97C11G220850	ko00010	1.00	3.73	phosphoenolpyruvate carboxykinase [ATP]
	Cla97C11G206570	ko00780	−0.47	−0.93	NADPH-dependent pterin aldehyde reductase

## Data Availability

The datasets had submitted to the National Center for Biotechnology Information (NCBI) repository, bioproject: PRJNA844416.

## Supplementary Materials

The following supporting information can be downloaded at: https://www.mdpi.com/article/10.3390/life12071033/s1, Figure S1: Phylogenetic tree of TCPs; Figure S2: Phylogenetic tree of TCPs; Table S1: Primers for q-PCR.; Table S2: Basic information about transcriptomic data; Table S3: Correlation coefficient between TFs and DEGs in roots; Table S4: Correlation coefficient between TFs and DEGs in leaves; Table S5: Correlation coefficient between TFs and DEGs in leaves.

## Abbreviations

RWC	relative water content
MDA	malondialdehyde acid
ROS	reactive oxygen species
NAA	naphthaleneacetic acid
JA	jasmonic acid
IAA	indole-3-acetic acid
GA	gibberellin
SA	salicylic acid
DEGs	differentially expressed genes
FDR	false discovery rate
ORF	open reading frame
TPS	trehalose-6-phosphate synthase
HKT	high-affinity K^+^ transporter
KUP	K^+^ uptake permease
PCA	principal component analysis
HSP	heat shock protein
CYP	cytochrome P450
SAUR	small auxin up RNA
GH3	gretchen hagen 3
ARF	auxin response factor
CESAs	cellulose synthases
PEPCK	phosphoenolpyruvate carboxykinase
