# Supplementary figures and images for: Comparative Transcriptome Profiling Provides Insights into Plant Salt Tolerance in Watermelon (Citrullus lanatus)
Source: Life (Basel). 2022 Jul 12;12(7):1033. doi: 10.3390/life12071033 (PMC9320501; doi:10.3390/life12071033)

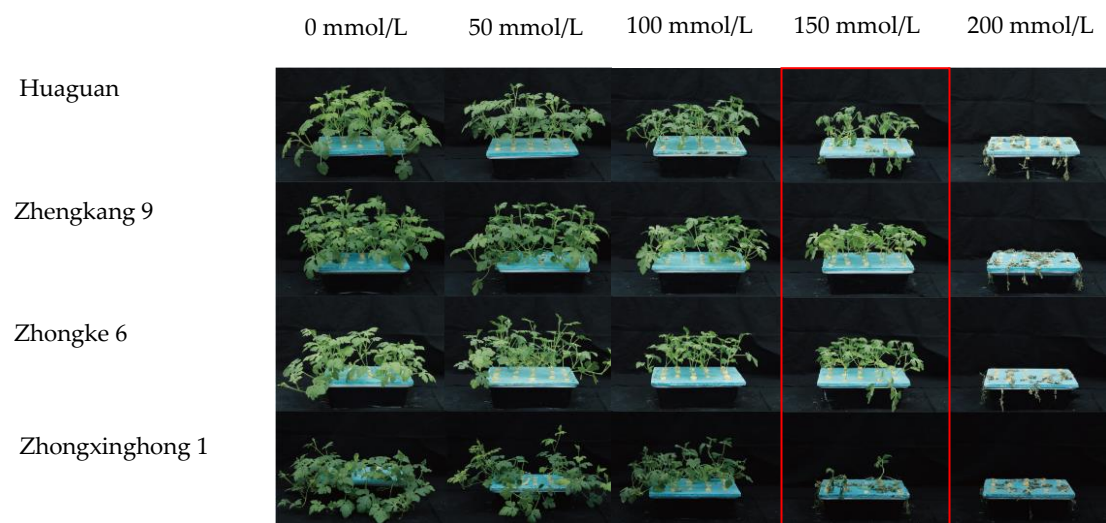

Figure S1. The salt tolerance of the different salt concentrations.

Supplement: Supplementary file 1 [file life-12-01033-s001.zip › supplementary Figure S1.pdf]

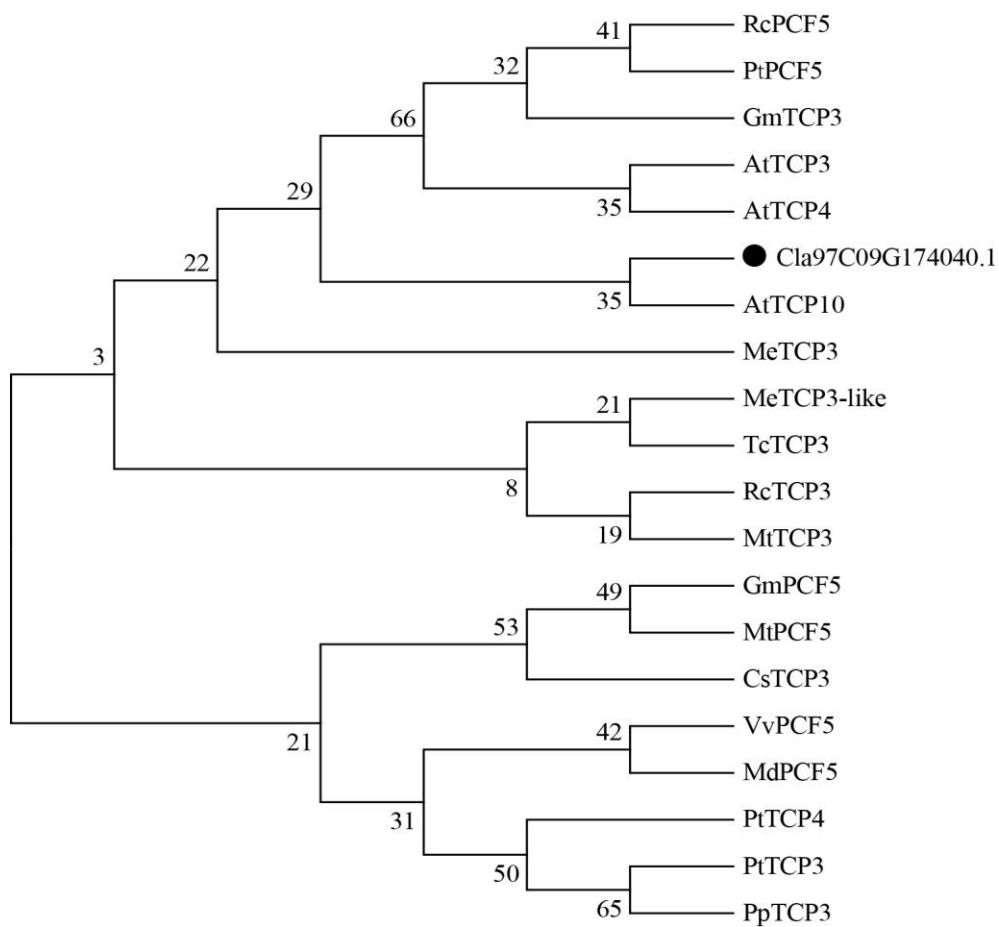

Figure S2. Phylogenetic tree of TCPs.

Supplement: Supplementary file 1 [file life-12-01033-s001.zip › supplementary Figure S2.pdf]
